# Two‐dimensional speckle tracking to image ventricular‐arterial coupling in uremia

**DOI:** 10.1111/echo.14183

**Published:** 2018-11-08

**Authors:** Fu‐Yong Ye, Yin‐Ting Liang, Xiao‐Chun Lin

**Affiliations:** ^1^ Department of Ultrasound The People's Hospital of Gaozhou City Gaozhou Guangdong China

**Keywords:** arterial elastance, end‐systolic elastance, left ventricular function, speckle tracking imaging, uremia, ventricular‐arterial coupling

## Abstract

**Objective:**

To study ventricular‐arterial coupling(VAC) in uremic patients by application of two‐dimensional speckle tracing imaging (2DSTI).

**Methods:**

One hundred uremic patients were divided into two groups based on left ventricular ejection fraction (LVEF): group 1 with LVEF ≥ 5%, and group 2 with LVEF < 55%. Forty healthy subjects were recruited as a control group. Conventional echocardiography was performed; VAC components and myocardial performance index were calculated. Longitudinal strain (LS) of 17 segments was measured using 2DSTI. Mean base (LS_BA_), papillary muscle (LS_PM_), and apex values (LS_AP_) were calculated.

**Results:**

Compared to subjects in the control group and group 1, subjects in group 2 exhibited decreased LV end‐diastolic volume (EDV), end‐systolic volume (ESV), LV mass index (LVMI), and VAC (*P *<* *0.05). EF, fractional shortening (FS), end‐systolic elastance (Ees) were significantly higher in group 2 (*P *<* *0.05). SL_BA_, SL_PM_, and SL_AP_ differed significantly among the groups (all *P *<* *0.05). SL_BA_, SL_PM_, and SL_AP_ correlated positively with Ees, EF, and FS (all *P *<* *0.05) but negatively with arterial elastance (Ea), VAC, systemic vascular resistance index (SVRI), and rate‐pressure product (RPP) (all *P *<* *0.05). Multiple regression analysis revealed that relative wall thickness (RWT), LVMI, LS_AP_, and stroke works (SW) were independent predictors of VAC (*b′* = −0.443, 0.537, −0.470, and −0.491, all *P *<* *0.05).

**Conclusions:**

In patients with uremia, LV myocardial LS gradually decreased as LV systolic dysfunction decreased. VAC correlated negatively with left ventricular LS, and LS_AP_ was an independent predictor for VAC.

## 1 INTRODUCTION

In recent years, a large body of evidence has identified cardiac dysfunction as a serious complication and the major cause of death in patients suffering from chronic uremia.^1^ The pathophysiologic and clinical implications of cardiac function should be considered together with vascular effects because ventricular‐arterial coupling (VAC), or the interaction between the left ventricle and the systemic artery, is an important determinant of cardiovascular performance.^2^, ^3^, ^4^

Two‐dimensional speckle tracking imaging (2DSTI) has been introduced as a feasible and reproducible technique with which to analyze regional and global left ventricular (LV) strain. This modality may be used to objectively assess myocardial deformation and quantitatively analyze regional wall motion abnormalities. 2DSTI is also preferable for evaluation of apical segment movement because the approach used avoids the angle dependence of Doppler technology.^5^, ^6^

The aim of this paper was to assess the utility of 2DSTI for evaluation of cardiovascular stiffness and VAC in patients with uremia.

## 2 MATERIALS AND METHODS

### 2.1 Study population

This study enrolled 100 patients (52 male, 48 female; mean age ± SD, 48.6 ± 12.8 years; range, 21–63 years) with uremia seen at the Urology Department of our hospital during the period from January 2015 to December 2017. Subjects were divided into 2 groups: group 1 included subjects with normal LV systolic function; group 2 included patients with LV systolic dysfunction. Systolic dysfunction was defined as LV ejection fraction (LVEF) <55%. Patients were excluded if they had valvular disease, coronary heart disease, hypertrophic cardiomyopathy, congenital heart disease, or peripheral arterial disease. All uremic patients were on hemodialysis for 4 hours a day, 3 times a week. Patients without standardized treatment were excluded. Each patient underwent assessment by 2DSTI before hemodialysis.

Forty healthy volunteers (20 male, 20 female; mean age, 44.80 ± 10.76 years; range, 22–65 years) who had no history of renal or cardiovascular disease were included as controls. All subjects included as controls had normal physical examination, echocardiographic, and electrocardiographic results. The study was approved by the Ethics Committee at Gaozhou People's Hospital, and each patient who participated in the study provided written informed consent.

### 2.2 Echocardiography

Two‐dimensional echocardiography was performed with a commercial ultrasound machine (iE33; Philips Healthcare, Andover, MA) equipped with an S5‐1 transducer (1–5 MHz). Conventional echocardiography was performed with the patient in the left lateral decubitus position. LV end‐diastolic diameter (EDD), LV end‐systolic diameter (ESD), LV posterior wall thickness (PWT), and interventricular septum thickness (IVST) were measured according to the criteria provided by the American Society of Echocardiography (Raleigh, NC). These parameters were used to calculate LV mass index (LVMI)^7^ and relative wall thickness (RWT).^8^ LV end‐diastolic volume (EDV) and end‐systolic volume (ESV), stroke volume (SV), and EF were measured with the apical biplane method. Cardiac output (CO) was obtained as SV × heart rate (HR), and cardiac index (CI) was derived from the ratio of CO to body surface area (BSA). All parameters above were measured in triplicate and averaged.

### 2.3 Ventricular‐arterial coupling

Arterial elastance (Ea) was estimated as end‐systolic pressure (ESP)/stroke volume (SV), with ESP estimated as systolic pressure × 0.9.

LV end‐systolic elastance (Ees) was calculated as follows:

$$\text{Ees}=\text{ESP}/(\text{ESV}-V_{0})\approx \text{ESP/ESV}$$

where *V*_0_ is LV volume when LV pressure = 0.

Accordingly, VAC was calculated as Ea/Ees.^9^

Stroke works (SW) was obtained as ESP × SV;^10^ rate‐pressure product (RPP) was obtained as HR × SBP;^11^ systemic vascular resistance index (SVRI) was obtained as mean arterial pressure (MAP) × 80/CI.^12^

### 2.4 2D speckle tracking analysis

Offline strain measurements were performed with Qlab 8.0 software. Apical four‐, two‐, and three‐chamber views were obtained with frame rates >60 frame/sec. The regional four‐chamber view of the left ventricular apex was analyzed first. Three points were selected for analysis. The software program automatically provided an outline of the endocardium to produce a region of interest (ROI). ROI width was adjusted manually to reflect myocardial thickness.^13^ The system automatically generated longitudinal strain values for 5 or 6 segments. The same method was used to analyze the data provided in apical two‐ and three‐chamber views. The left ventricle was divided in accordance with the American Society of Echocardiography's 17‐segment model. A bull's eye display of longitudinal peak strain was obtained for all segments of the left ventricle. Mean longitudinal strain (LS) of myocardial segments including the base (LS_BA_), papillary muscle (LS_PM_), and apex (LS_AP_) were calculated.^14^

### 2.5 Statistical analysis

Statistical analyses were performed using SPSS 20.0 software (SPSS Inc, Chicago, IL). All data are expressed as mean values ± SD. Analysis of variance for multiple comparisons was used to assess between‐group differences. Pearson's correlation coefficients were applied to evaluate correlations between variables of interest. Multiple linear regression analysis was performed to explore the determinants of VAC. *P *<* *0.05 was considered statistically significant.

## 3 RESULTS

### 3.1 Clinical characteristics

Descriptive characteristics of the patient population are presented in Table 1. There were no significant differences among the 3 groups in terms of age or gender. However, systolic blood pressure (SBP), mean arterial pressure (MAP), and heart rate (HR) were higher in the 2 groups with uremia than in the control group. Body mass index (BMI) was lower in the 2 groups with uremia than in the control group (*P *<* *0.05) (Table 1).

**Table 1:** Clinical parameters of the study population

Parameter	Control (n = 40)	Group 1 (n = 50)	Group 2 (n = 50)	*F*	*P*
Age, y	44.80 ± 10.76	47.50 ± 11.57	49.6 ± 14.01	1.695	0.187
Male/female	20/20	26/24	26/24	—	0.974
BMI, kg/m^2^	22.74 ± 2.63	21.09 ± 3.35^a^	21.24 ± 2.48^a^	4.353	0.015
SBP, mmHg	127.70 ± 17.07	149.32 ± 22.33^a^	152.72 ± 24.34^a^	16.721	0.000
MAP, mmHg	96.52 ± 13.70	109.87 ± 16.20^a^	110.73 ± 18.61^a^	10.045	0.000
HR, bpm	79.35 ± 9.63	83.92 ± 7.63^a^	84.04 ± 8.73^a^	4.115	0.018

Values (except male/female) are presented as mean ± SD.

BMI = body mass index; SBP = systolic blood pressure; MAP = mean arterial pressure; HR = heart rate.

a *P *<* *0.05 vs control.

### 3.2 Conventional echocardiographic parameters and LV longitudinal strain

**Figure 1:**
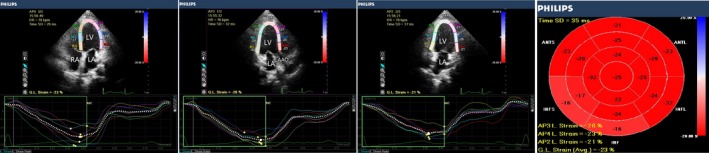
2D longitudinal peak strain, presented as a “bull's eye” display. Representative image of a control subject

**Figure 2:**
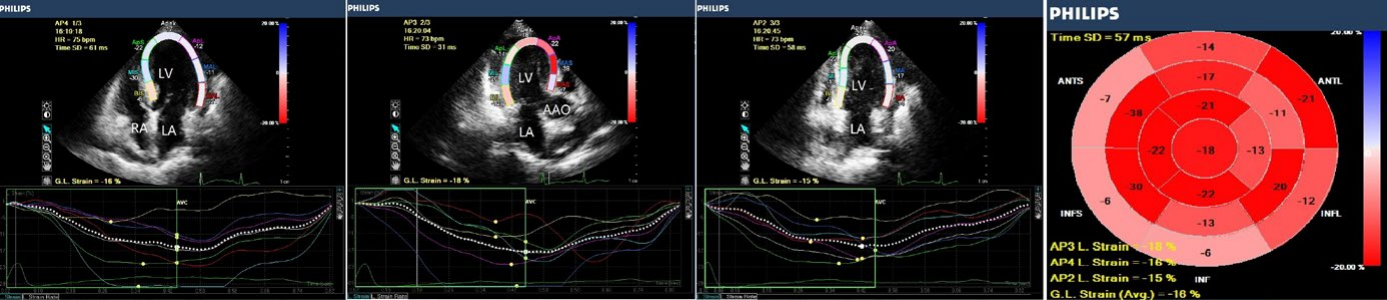
2D longitudinal peak strain, presented in a “bull's eye” display. Representative image from a uremic patient with normal LV systolic function

**Figure 3:**
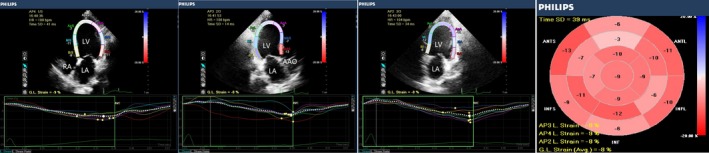
2D longitudinal peak strain, presented as a “bull's eye” display. Representative image from a uremic patient with LV systolic dysfunction

RWT and LVMI were higher in group 1, compared with the control group (*P* < 0.05). LS_BA_, LS_PM_, and LS_AP_ were lower in group 1 (*P *<* *0.05), compared with the control group. LVEDV, LVESV, and LVMI were significantly higher in group 2, compared with the control group and group 1 (*P *<* *0.05). EF, FS, LS_BA_, LS_PM_, and LS_AP_ were significantly lower in group 2 than in the control group and group 1 (*P *<* *0.05). RWT was slightly higher in group 2 than in the control group, but this trend was not statistically significant. There were no differences across groups in SV, CO, or CI (Table 2, Figures 1, 2, 3).

**Table 2:** Results of conventional echocardiography vs. speckle tracking imaging

Parameter	Control (n = 40)	Group 1 (n = 50)	Group 2 (n = 50)	*F*	*P*
LVEDV, mL	85.47 ± 15.56	93.61 ± 27.32	129.14 ± 33.08^a^, ^b^	34.864	0.000
LVESV, mL	28.31 ± 6.88	33.28 ± 10.31	71.45 ± 26.50^a^, ^b^	87.457	0.000
SV, mL	57.16 ± 10.90	60.33 ± 18.63	57.69 ± 15.22	0.564	0.570
CO, L/min	4.56 ± 1.02	5.11 ± 1.57	4.87 ± 1.44	1.761	0.176
CI, L·min^−1^·m^−2^	2.68 ± 0.58	3.09 ± 0.87	2.93 ± 0.83	3.047	0.051
EF,·%	66.85 ± 4.72	64.4 ± 4.68	45.74 ± 9.90^a^, ^b^	128.1	0.000
FS,·%	36.92 ± 3.63	35.13 ± 3.45	23.23 ± 5.72^a^, ^b^	133.313	0.000
RWT	0.43 ± 0.04	0.56 ± 0.12^a^	0.47 ± 0.09^b^	23.044	0.000
LVMI,·g/m^2^	78.50 ± 11.27	127.84 ± 32.63^a^	149.69 ± 33.86^a^, ^b^	69.944	0.000
LS_BA_,·%	17.08 ± 1.51	14.12 ± 2.63^a^	11.18 ± 2.30^a^, ^b^	77.279	0.000
LS_PM_,·%	20.53 ± 1.75	17.32 ± 2.71^a^	13.46 ± 2.39^a^, ^b^	101.306	0.000
LS_AP_,·%	24.65 ± 1.08	21.88 ± 2.97^a^	14.32 ± 2.85^a^, ^b^	207.284	0.000

Data are presented as mean ± SD.

LVEDV = left ventricular end‐diastolic volume; LVESV = left ventricular end‐systolic volume; SV = stroke volume; CO = cardiac output; CI = cardiac index; FS = fractional shortening; RWT = relative wall thickness; LVMI = left ventricular mass index; LS_BA_ = longitudinal strain at the level of the base; LS_PM_ = longitudinal strain at the level of papillary muscles; LS_AP_ = longitudinal strain at the level of the apex.

a *P* < 0.05 vs control group.

b *P* < 0.05 vs group 1.

### 3.3 VAC components and myocardial performance index

With respect to VAC, Ea was significantly higher in group 2, compared with the control group. Ea values in group 2 were not significantly different from those reported for group 1. Ees and VAC values for group 2 differed significantly from those observed for the control group and group 1. Ees and VAC values were similar in the control group and group 1.

RPP and SW were significantly higher in the 2 disease groups, compared with the control group. SVRI did not differ significantly among the 3 groups (Table 3).

**Table 3:** VAC components and myocardial performance index

Parameter	Control (n = 40)	Group 1 (n = 50)	Group 2 (n = 50)	*F*	*P*
Ea, mmHg/mL	2.08 ± 0.45	2.43 ± 0.81	2.58 ± 0.95^a^	4.677	0.011
Ees, mmHg/mL	4.32 ± 1.27	4.45 ± 1.57	2.22 ± 0.99^a^, ^b^	44.889	0.000
VAC (Ea/Ees)	0.50 ± 0.11	0.56 ± 0.11	1.32 ± 0.66^a^, ^b^	61.633	0.000
RPP·mmHg·bpm	10234 ± 2095	12760 ± 2534^a^	12858 ± 2595^a^	15.92	0.000
SW·mmHg·mL	6601 ± 1645	8104 ± 2678^a^	7914 ± 2342^a^	5.431	0.005
SVRI·N·m^2^·cm^−5^	0.030 ± 0.008	0.031 ± 0.011	0.033 ± 0.012	0.923	0.400

Data are presented as mean ± SD.

Ea = arterial elastance; Ees = end‐systolic elastance; VAC = ventricular‐arterial coupling; RPP = rate‐pressure product; SW = stroke works; SVRI = systemic vascular resistance index.

a *P* < 0.05 vs control group.

b *P* < 0.05 vs group 1.

### 3.4 Correlation analysis

Pearson correlation analysis was conducted to investigate the relationship between LV longitudinal strain and conventional cardiovascular parameters. For the overall study population, LS_BA_, LS_PM_, and LS_AP_ were positively correlated with Ees, EF, and FS (all *P *<* *0.05) and negatively correlated with Ea, VAC, SVRI, and RPP (all *P *<* *0.05). No significant correlation with SW and RWT was observed. Multiple linear regression analysis showed that RWT, LVMI, LS_AP_, and SW were independent predictors of VAC. Standard regression coefficient *b′* was −0.443, 0.537, −0.470, and −0.491, respectively (all *P *<* *0.05) (Tables 4 and 5).

**Table 4:** Correlation of LV strain with cardiovascular variables

Parameters	LS_BA_	LS_PM_	LS_AP_
Ea	−0.36^a^	−0.323^a^	−0.348^a^
Ees	0.385^a^	0.452^a^	0.561^a^
VAC	−0.623^a^	−0.643^a^	−0.76^a^
SVRI	−0.226^a^	−0.185^a^	−0.234^a^
SW	0.051	−0.079	0.023
RPP	−0.397^a^	−0.359^a^	−0.285^a^
RWT	−0.123	−0.135	−0.033
LVMI	−0.495^a^	−0.567^a^	−0.545^a^
EF	0.683^a^	0.707^a^	0.84^a^
FS	0.69^a^	0.707^a^	0.843^a^

a *P* < 0.05.

**Table 5:** Multiple linear regression analysis to determine VAC in uremic patients

Variable	Regression coefficient (*b*)	Standard error (*Sb*)	Standard regression coefficient (*b′*)	*t*‐value	*P*‐value
Constant	2.989	0.194		15.385	0.000
RWT	−2.377	0.270	−0.443	−8.812	0.000
LVMI	0.007	0.001	0.537	7.454	0.000
LSAP	−0.051	0.006	−0.470	−8.104	0.000
SW	0.000	0.000	−0.491	−8.118	0.000

## 4 DISCUSSION

The principal finding of this observational study was that in patients with uremia, mean longitudinal strain accurately reflects arterial stiffness, myocardial performance, and VAC. In patients with uremia, LV myocardial LS decreased gradually as LV systolic dysfunction decreased. VAC is normal in uremic patients with normal LV systolic function but abnormal in those with LV systolic dysfunction. VAC correlated negatively with left ventricular LS, and LS_AP_ was an independent predictor of VAC.

Cardiovascular‐related issues are an important cause of death among patients with uremia. Chronic renal disease is associated with a high incidence of changes in cardiovascular morphology and function. Chronic renal disease is caused by several factors^15^: Hypertension may result in an enlarged heart, a thickened ventricular wall, and increased myocardial oxygen consumption. Chronic renal disease may also accelerate the process of arterial stiffening. Secondly, chronic hypervolemia may negatively impact the ventricular myocardium. Chronic hypervolemia may result in increased ventricular preload, increased oxygen requirement, and a stepwise increase in cardiac insufficiency. Finally, chronic anemia may lead to myocardial ischemia and hypoxia in uremic patients. Electrolyte imbalances may damage the myocardium, resulting in cardiac failure. Previous studies of cardiovascular disease in uremic patients have shown that age is a risk factor for cardiovascular‐related death.^16^ There was no significant difference in age or gender among the three groups included in our study, demonstrating that the factors above did not affect the research results.

With respect to components of the VAC, Ea is an integrated index of the net arterial load imposed on the LV. Numerous disease states are characterized by elevated Ea.^10^ Ees is a measure of myocardial contractility reflecting the ability of the LV to eject blood, in opposition to a given pressure. An increase in Ees is usually associated with enhanced myocardial contractility. In addition, chronic changes in Ees also reflect passive myocardial stiffening and chamber geometry.^17^ VAC, a crucial determinant of cardiovascular performance, is reliably estimated by the ratio of Ea to Ees. Whereas studies of normal healthy subjects have shown optimal coupling at VAC values close to unity, the ventricle achieves maximal efficiency when VAC approaches 0.5.^18^, ^19^

In uremic patients, vascular sclerosis increases myocardial oxygen; myocardial stiffness further enhances this effect. Table 3 shows that RPP, a sensitive index of myocardial oxygen consumption, increased remarkably in uremic patients, as did SW, an index of myocardial performance. As the disease progresses, cardiovascular reserve decreases, with accompanying effects on the ventricular‐vascular interaction.^19^, ^20^ Increasing cardiac insufficiency induces stepwise deterioration of VAC. In this study, increased Ea and decreased Ees were observed in subjects with LV systolic dysfunction (group 2). VAC (Ea/Ees ratio) was <1.0 in subjects with normal LV systolic function (group 1). VAC was similar in group 1 and in the control group. In group 2, VAC was >1.0. This value is significantly higher than that observed in the control group or group 1.

2DSTI technology is a noninvasive tool that has recently garnered increasing interest among clinicians. This modality provides a quantitative analysis of segmental contractility and an objective evaluation of myocardial function.^21^, ^22^, ^23^ In our study, mean SL in two groups with uremia (group 1 and group 2) in the short‐axis view (LS_BA_, LS_PM_, and LS_AP_) was significantly decreased, especially in patients with LV systolic dysfunction (group 2). Correlation analysis revealed a moderate negative correlation between VAC and LS_BA_, LS_PM_, and LS_AP_ (in all participants). Ees was positively correlated with LSBA, LSPM, LSAP; Ea was negatively correlated with LS_BA_, LS_PM_, and LS_AP_.

Previous studies have investigated the determinants of VAC in hypertension and diastolic dysfunction.^24^, ^25^ In contrast to previous reports that neither LS_BA_ nor LS_PM_ shows changes in response to hypertensive stimuli, LV contractility is affected mainly by LS_AP_ rather than LS_BA_ or LS_PM_, suggesting that LS_AP_ may contribute to VAC only partially.^24^, ^25^ Our results are consistent with these findings: changes in LS_AP_ are a major determinant of VAC, whereas changes in LS_BA_ and LS_PM_ may have only minor effects on VAC.

## 5 LIMITATIONS

This study had some limitations. Firstly, the duration of uremia should be an important parameter when analyzing the effects of uremia on cardiac function and VAC. However, this study failed to analyze the duration of illness in uremic patients, as some patients did not know how long they had been suffering from uremia. Secondly, during dialysis treatment, blood biochemical parameters associated with uremia changed dynamically. This study did not include laboratory analysis. It is expected that in future studies, changes in cardiac function and VAC during this dynamic process will be analyzed. Finally, future studies should investigate global LS in uremic patients.

## 6 CONCLUSION

In conclusion, LV myocardial LS decreased gradually as LV systolic dysfunction decreased in patients with uremia. VAC was normal in uremic patients with normal LV systolic function but abnormal in patients with LV systolic dysfunction. VAC correlated negatively with left ventricular LS, and LS_AP_ was an independent predictor for VAC.
